# Sir F. Treves and Parliament

**Published:** 1903-07-25

**Authors:** 


### EDITOR'S LETTER-BOX.

**[Our Correspondents are reminded that prolixity is a great bar to publication, and that brevity of style and conciseness of statement greatly facilitate early insertion.]**

### SIR F. TREVES AND PARLIAMENT.

SIR F. TREVES writes: I am much obliged to you for your very kind reference to me in the leading article of THE HOSPITAL for July 11th. I may say that I have no intention of endeavouring to enter Parliament, but your most encouraging remarks would almost induce one to alter that decision.

